# A Review of Success Factors for Piglet Fostering in Lactation

**DOI:** 10.3390/ani8030038

**Published:** 2018-03-09

**Authors:** Jena G. Alexopoulos, David S. Lines, Suzanne Hallett, Kate J. Plush

**Affiliations:** SunPork Farms, P.O. Box 92, Wasleys 5400, SA, Australia; david.lines@sunporkfarms.com.au (D.S.L.); suzanne.hallett@sunporkfarms.com.au (S.H.); kate.plush@sunporkfarms.com.au (K.J.P.)

**Keywords:** colostrum, split suckling, udder assessment, rearing ability, nurse sow, piglet survival

## Abstract

**Simple Summary:**

An understanding of behavioural and physiological mechanisms responsible for piglet survival and growth will assist in developing the best recommendations in which to manage piglet movements in the farrowing house. This review has identified six key principles that should underpin successful piglet fostering. These fostering principles will improve productivity and welfare of sows and piglets in commercial pig production.

**Abstract:**

Piglet movement from one sow to another, or fostering, is required in modern pig farming but there is little available literature on the most effective strategy. In this review, we focus on the behavioural and physiological mechanisms responsible for piglet survival and growth, and have identified six key principles. (1) Colostrum provides piglets with warmth, energy and immunity. It is most accessible during the first 12 h from the birth sow, therefore no piglet should be moved before this; (2) To ensure even intake of birth sow colostrum, techniques such as split suckling prior to piglet movement should be implemented; (3) Udder assessment for functional teats should occur at farrowing, with number of fostered piglets not exceeding teat number; (4) Primiparous sows should receive as many piglets as the udder allows to maximise mammary stimulation, although older parities should be assessed for rearing ability; (5) Piglet fostering should occur between 12 and 24 h and movement kept to a minimum to prevent transfer of disease; Litter outliers should be moved and relocated to a litter of similar size; (6) Piglet movement after 24 h should be minimised. When required, strategies such as nurse usage should be employed. These principles will result in improved farrowing house performance by increasing the litter weight weaned per sow.

## 1. Introduction

Piglet movement from one sow to another, known as fostering, is done frequently when the number of piglets a sow gives birth to does not match her rearing ability. Circumstances may also arise where piglet relocation is required such as sow illness or death, or when a piglet fails to thrive on their birth sow. There are few available published data that make recommendations as to the best way in which to manage piglet movement during lactation. This is most likely because it may be foolish to accept that a “one size fits all” approach would be suitable given the large variability in which farrowing barns are managed across herds. In this review we focused on both the sow and piglet physiological and behavioural influences that are important for litter survival and growth. This should then assist producers to make informed decisions on how best to move piglets within each production system. The structure follows a chronological order of events that should be followed from parturition through to weaning.

## 2. The Importance of Colostrum

### 2.1. What Is Colostrum?

**Figure 1:**
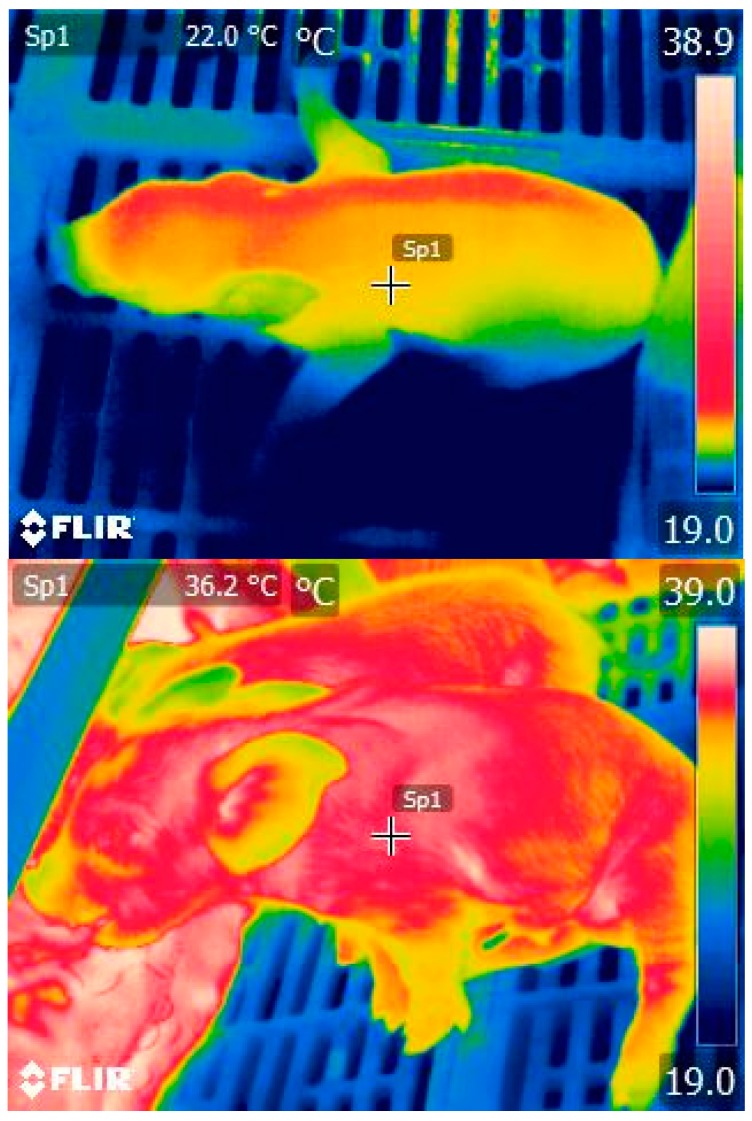
Thermal image detecting skin temperature of newborn piglets that are receiving colostrum (lower: 36.3 °C), and of a “low viability” piglet who has failed to reach the udder and so ingest colostrum (upper: 22.0 °C). Thermal colour scale (19–39 °C) presented on the right hand-side of each image. Image taken by Jena G. Alexopoulos.

At birth piglets are exposed to an abrupt change in energy supply as they begin enteral feeding. Additionally, the environment a piglet is born into is generally cold. Heat is rapidly lost from the newborn piglet because of a high surface area to volume ratio and wet skin and so body temperature declines rapidly [1]. Newborn piglets have less than 2% body fat but have high energy requirements and so enter a negative energy balance shortly after birth [1]. Colostrum is composed of protein, fat and carbohydrates, all of which are energy rich [2] and help piglets to overcome this negative energy balance. Thus, finding a teat is crucial for piglet survival [3]. Colostrum composition differs significantly to the milk that follows in that it has higher concentrations of dry matter and crude proteins, but lower concentrations of lactose and fat. However, the fat present in colostrum still provides piglets with 40–60% of their total energy supply [2]. As a piglet begins to suck and ingest colostrum energy and warmth are provided which act to increase body temperature and viability (Figure 1), both being strongly linked to survival [4,5].

**Figure 2:**
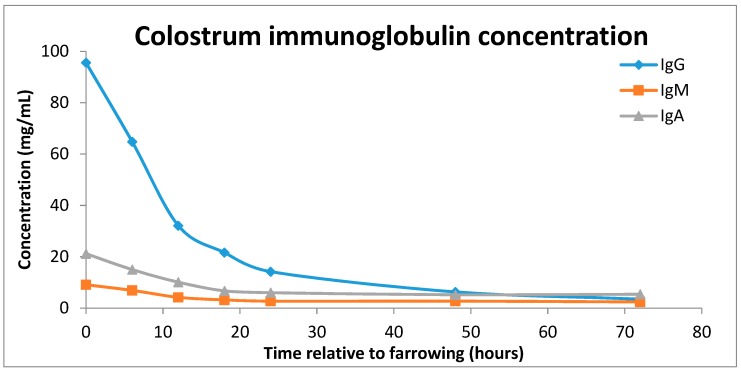
Concentration of immunoglobulins (IgG blue line, IgM, orange line and IgA, purple line) in sow colostrum over a 72 h period post farrowing (adapted from Klobasa, Werhahn and Butler [7]).

In addition to these major changes in energy utilisation, piglets are born immunologically naïve as the sow is unable to transfer antibodies in utero to the piglet via the placenta [6]. Thus, antibody transfer from colostrum is crucial for adequate immune function. Immunoglobulin (Ig) G is the predominant antibody in colostrum and acts to protect the piglet against infections. Colostrum also contains IgA and IgM, leukocytes, selenium and vitamin E, all of which are important for immune function [2]. The concentrations of colostral IgG are several-fold higher at parturition than in sow plasma but decrease rapidly over the first 24 h (Figure 2; Klobasa et al. [7]). Milk IgG concentration is relatively low when compared with colostrum, with IgA being the dominant immunoglobulin after transition to milk. IgA protection acts at mucosal surfaces, including respiratory and gastrointestinal tracts [5]. The importance of colostrum for piglet immunity has been reviewed thoroughly previously [2,5] and so no more detail will be given here.

Colostrum is best derived from a piglet’s own birth sow. Tuboly et al. [8] showed improvements in immune cell absorption through the intestinal barrier from birth sow colostrum compared with non-birth sow donors. More recently, Bandrick et al. [9] reported that there were some aspects of immunity present in colostrum (cell mediated immunity) detected in piglets only when they were maintained on their birth sow for greater than 12 h. Thus, to optimise the degree of maternal immunity transferred via colostrum, the piglet should be maintained on their birth sow for at least 12 h.

### 2.2. Impacts of Colostrum Intake

Colostrum intake is one of the main determinants of piglet survival [10]. Quesnel et al. [11] reported that piglets who received more than 200 g of colostrum displayed a 7.1% mortality rate, but those receiving less than 200 g were six times (43%) more likely to die during the sucking stage. The effects of colostrum intake on mortality are evident after weaning also. Varley et al. [12] reported that when weaned from the sow immediately following birth (i.e., did not receive colostrum) only 25% of pigs survived to two weeks, under 30% of those pigs made it to six weeks, and no pigs survived to 90 kg. Pre-weaning mortality of piglets with low and average birth weights, which are traditionally cited as having increase risk of death, can achieve similar survival to heavier counterparts when colostrum intake reached between 200 g and 250 g [13]. Similarly, growth restricted piglets show improvements in traits important for survival when colostrum is administered [14]. Thus, ensuring that small piglets receive adequate colostrum improves survival.

In addition to survival, there are other long-term benefits associated with increased colostrum intake. Improved growth and high weaning weights have been reported in piglets receiving high quantities of colostrum. Greater than 290 g of colostrum ingestion resulted in an increase in weight of roughly two kilograms at six weeks of age when compared with those piglets who consumed less than 290 g [15]. There would be obvious health improvements in these piglets that may explain these findings, but piglets that absorb high amounts of colostrum also display improvements in small intestinal maturity. Colostrum intake improves the development of mucosal brush border enzymes increasing lactose and maltose metabolism in the first 24 h after birth [16,17]. When fed to 28 days of age, piglets reared on a colostrum isolate ate more, had greater gut weight, grew faster, and showed greater concentrations of IGFs 1 and 2 than those fed a whey based concentrate [18], confirming the impacts on tissue growth. It has been suggested this improvement in intestinal development could positively influence feed conversion later in life [5].

Whilst vaccines are an effective way to manage post-weaning piglet health, prior to their use colostrum intake reduced disease susceptibility in the post-weaning period. Once a piglet is withdrawn from sow milk (i.e., weaned) they become highly susceptible to enteric diseases because of the altered balance between the development of microbiota and establishment of intestinal bacterial pathogens [19]. A review by Allan et al. [20] reported a higher degree of susceptibility for porcine multi-systematic wasting syndrome after weaning in piglets that were deprived of colostrum compared to piglets that were fed colostrum. Similarly, Elbers et al. [21] demonstrated that herds with porcine multi-systematic wasting syndrome had poor colostrum intake by piglets compared with those with no incidence of the disease.

The maternal rearing environment can exert permanent influences on the offspring, a process known as maternal programming. In the piglet, there is evidence that the colostrum and milk of the sow signals reproductive tissue growth and maturation. Female piglets artificially reared on milk-replacer in the absence of colostrum showed altered expression of genes responsible for endometrium growth and delayed uterine gland development [6]. Subsequently, Vallet et al. [22] reported an increased age at puberty and reduction in number of piglets born when estimated colostrum intake was low. Additionally, this study showed longer term effects with piglets born to sows that ingested low colostrum amounts as neonates exhibiting decreased growth rates and low blood serum concentrations of Igs early in life.

### 2.3. Sow Colostrum Production

Colostrum production in the mammary gland occurs prior to the birth of the first piglet and therefore is not dependent on litter size [1]. Colostrum production has been reported to range from 2.5–5 kg [23]. Both colostrum quality and quantity can be manipulated genetically, hormonally and nutritionally [24]. Colostrum is secreted from the udder immediately at farrowing [25] and is continuously supplied to piglets from farrowing for approximately two to four hours, thereafter it is released in more discrete letdowns [26,27]. Colostrum production is highest at farrowing but decreases rapidly to 14 h, and by 34 h copious quantities of transitional milk is produced [1]. In comparison, colostrum yield is lower in gilts than in sows [28]. Additionally, IgG concentrations are significantly lower in gilt colostrum [29] and decreases more rapidly over the first 24 h [30] when compared with that from sows. Thus, gilt litters may not receive the same quantity and quality colostrum than those from sows. Indeed, Lines [31] showed that piglets from gilt litters displayed reduced colostrum intake compared to those from sow litters.

### 2.4. Piglet Colostrum Ingestion

When fed artificially, piglets will consume double the amount of colostrum ingested under sow-rearing conditions, indicating that the sow typically limits the colostrum intake of her piglets [32]. This represents a larger issue in more recent times as the selection towards increased litter size has had little impact on colostrum production of sows [28,30]. Intake of colostrum is heavily dependent on the piglets ability to reach the teat, with viability being the most likely factor responsible for the variability in ingested colostrum between piglets (0–700 g) [11]. In addition to this, piglets with low birth weights born into large litters with average to large litter mates tend to miss more nursing episodes and spend more time in teat disputes [33], therefore have a reduced intake of colostrum. Piglets with congenital defects such as splayed legs are also disadvantaged and ingest less colostrum. They are much slower to their feet and to the udder, which compromises their ability to compete with litter mates shortly after birth [25,28]. Given this, these “at risk” piglets may require intervention to achieve an adequate colostrum intake.

Studies examining the impact of birth order intake of colostrum ingestion are contradictory. Devillers et al. [28] reported no difference in piglet weight gain in the first 24 h irrespective of their birth order. The authors explained that piglets spend a third of their first day nursing [27], and that generally three quarters of piglets are present at a nursing event during this first day [34]. Thus, it is likely that the later born piglets receive adequate colostrum intake whilst the earlier born piglets are sleeping. This study did record a numerical decrease in colostrum intake in the last-born category which was not statistically significant, most likely because piglet numbers in this category were low. Devillers et al. [15] reported that piglets born later in the farrowing process had lower serum concentrations of IgG suggesting colostrum intake was reduced, agreeing with previous reports [7]. At first appearance, these studies would contrast one another but in fact, declining colostral IgG concentrations may be used to explain discrepancies. Piglets born earlier in the birth order would have access to colostrum with higher concentrations of Igs than those born later. Thus, whilst actual piglet colostrum consumption in terms of weight may not alter with birth order, the quality of colostrum will. Therefore, piglets that are born late in the birthing order are at risk of inadequate acquired immunity through consumption of lower quality colostrum.

Piglet ability to absorb antibodies from colostrum begins to rapidly decrease after six hours from first feeding due to gut closure initiated by the presence of nutrients in the gut and their absorption [5]. As gut permeability decreases large proteins are unable to be absorbed and so the Ig’s required for passive immunity can no longer cross gut membranes [35]. Gut closure is complete in sucking piglets as early as 24 h of age [5]. This is an important point, as not only does sow colostrum production decline rapidly after farrowing, the piglets ability to absorb proteins important for immune function also decreases.

### 2.5. Ensuring Colostrum Absorption Prior to Fostering

Providing adequate colostrum is an industry challenge due to variation in colostrum production between sows and intake between piglets. It has been suggested that increasing litter sizes will reduce colostrum consumption in individual piglets thus prolificacy should be adjusted to the colostrum production capacity, and colostrum stores should be kept on farm to assist feeding [15]. Increased sow prolificacy may act to increase the variability of colostrum intake of piglets within a litter therefore practices that ensure even access of colostrum across piglets should be exploited. The process of split suckling ensures the supply of colostrum is distributed more evenly across a litter eventually acting to reduce variability in litter weight and increase chance of survival [36]. It involves removing larger, first born piglets from the udder for several milk let-down events giving the smaller, later born piglets multiple sucking sessions without the competition of larger litter mates [37,38]. In order to be effective, split suckling should occur as close to farrowing as possible and prior to 24 h post farrowing in order to achieve maximal colostrum intake in “at risk” individuals [25]. A two-hour session would allow two to three milk-let down events for the “at risk” piglets whilst balancing any potential impacts on the piglets removed from the sow. A behavioural study identified that lighter piglets did not exploit the reduction in competition [39] and so may benefit from stockperson assistance (re-warming, being placed on udder and assisted to suck) during the split suckling event. Subsequent studies have shown that split suckling results in improvements in survival. Vallet [40] reported increased survival rates and increased serum protein values of small piglets when split suckling was implemented. Similarly, Huser et al. [41] showed that the survival of small piglets was improved by 13% in split suckled litters. Other ways of ensuring adequate colostrum intake of piglets is through supplementation of either foreign sow colostrum [42] or artificial (usually bovine derived) colostrum products [43]. Both will be inferior substitutes for birth sow colostrum and so should only be utilised when necessary (such as sow death).

## 3. Assessment of Sow Rearing Ability Prior to Farrowing

### 3.1. The First Lactation

The activation of mammary tissue by piglet suckling in first lactation sows has been linked to milk output in the next lactation. Piglets from teats suckled in a previous lactation gained more weight, so that by eight weeks of age they were over a kilogram heavier than those piglets who suckled from a teat unused in the previous lactation [44]. This study also identified a higher weight of functional mammary tissue in glands previously suckled than those not, as well as behavioural differences in the piglets suggesting that piglets sucking from previously un-suckled teats were hungrier. A subsequent experiment indicated that a teat did not need to be stimulated for longer than two days for the improvement in a following lactation to be significant [45]. Thus, if a first parity sow is loaded to teat capacity for the first two days, the improvement in subsequent milk production should be evident even in the event of piglet death or removal later in lactation.

If recommendations are to be made from the above-mentioned research, a first parity sow must have all teats activated by piglets to maximise milk production as a parity two sow. However, there is a notion that gilts should receive fewer piglets at fostering for two reasons. Firstly, it was thought that due to reduced milk output, a gilt cannot wean as many piglets as older parity sows. However, a recent report containing data from a large commercial piggery identified that piglet survival did not differ between gilt and sow litters when managed appropriately (89% versus 90% [31]). Secondly, the suckling load of first parity sows may influence subsequent rebreeding success [46] as they can be more sensitive to weight loss in lactation than older parities [47]. However, there are strategies outside the scope of this review than can be employed to manage this issue. Thus, to maximise lifetime milk output, first parity sows should be allocated as many piglets as functional teats (explored shortly), but monitored for instances of poor rebreeding.

### 3.2. Repeatability of Number of Piglets Weaned, and Weaning Weight

**Figure 3:**
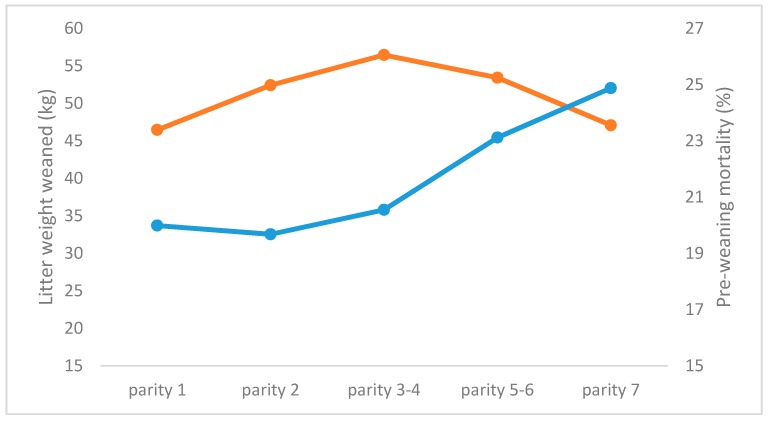
Litter weight at weaning (kg, orange line) and pre-weaning mortality (%, blue line) for sows with increasing parity (adapted from [48]).

In addition to first lactation sow management, any fostering decision should also pay attention to older sows. Both piglet survival and litter weaning weight decline after the sow reaches her third or fourth parity [48]. This notion is supported by Zhang et al. [48] which both survival of pigs and litter weight at day 21 of age decease with older ages (Figure 3). The likely explanations for poor piglet survival are increased sow disease pressure acting to reduce milk output [49,50], over-conditioning, udder damage [51] and poor teat accessibility [52]. Additionally, older sows are more prone to lameness [53] and this can increase the risk of piglet crushing [54,55], resulting in a higher mortality rate. Cytokines released associated with lameness act to reduce feed intake [56,57]. To support this notion, a negative relationship between dry-matter intake and locomotion score has been observed in other species [58]. Taken collectively, older sows are more prone to rearing fewer piglets. 

There is often debate as to whether the individual sow’s previous rearing ability should be considered at fostering. There is evidence that some sows crush more piglets than expected by chance, and some individual sows showed consistency across parity in the crushing of piglets [59]. This is partially supported by the finding that litter weight weaned has some degree of repeatability (0.27) [60]. However, this repeatability of rearing ability is low in sows and so should not receive too much attention with regards to fostering.

## 4. Assessment of Udders

### 4.1. Characteristics of a Functional Teat

A functional teat is defined as a mammary gland that successfully produces enough milk to rear a piglet. Conversely, a non-functional teat can be described as inverted, blind or small/extra teat that cannot be suckled, or a mammary gland that produces a reduced amount of milk limiting rearing capacity and increasing vulnerability to mastitis [61]. Chalkias et al. [62] showed clearly that piglets nursing from inverted teats cannot survive. Classifying a teat as functional can be performed at any time by visual inspection [63], but more certainty could be attained if counting occurs at farrowing, or if a teat is manipulated around farrowing to ensure production of milk.

### 4.2. What Are the Benefits of Counting Functional Teats

The number of functional teats available per piglet is positively associated with piglet survival. Where piglets had access to less than one functional teat mortality was greater than 14%, and when more than one teat was available it was reduced to below 8% [64]. This would suggest that fostering should take into account the number of functional teats available, and at a minimum, one teat should be allocated to each individual piglet. In fact, there is evidence that even when one teat per piglet is allocated, a piglet misses out on the milk let-down event for a given nursing [52]. The authors suggest this may be due to some dominant piglets monopolising more than one teat. Perhaps to buffer this scenario, the last caudal teats should not be taken in to account when counting functional teats as then outcompeted piglets may still access a gland during milk letdown. Additionally, rear teats have reduced mammary tissue resulting in lowest piglet weight gain [65], and may not be adequately presented during milk down. Thus, for fostering purposes, rear teats should not be counted as functional.

### 4.3. Other Udder Morphology of Importance

Piglets tend to suck first from teats that are close to the abdominal midline and have longer inter-teat distances [66], which would suggest that not only the number of teats but also positioning for those teats are important for access. On younger parity sows proximity to the midline makes teats more accessible, but with older parities larger udders may make teat access for piglets more difficult. Confirming this, Vasdal and Andersen [64] identified that the use of the lower teat row was reduced in multiparous sows, and this resulted in lower weight gains over the first 24 h in piglets from high parity (three to five) sows. This study also indicated that piglet number and weight at weaning was negatively affected by the diameter of a sows teat, suggesting that larger teats are more difficult to suck and so result in increased piglet mortality and decreased growth.

### 4.4. Making Progress in Udder Characteristics

Reports of greater than 15 functional teats are being cited from European studies [67], suggesting that herds should focus on improvements in udder traits. The heritability of functional and non-functional teats at 100 kg live weight was reported as 0.42 and 0.29, respectively [62], and so good genetic progress can be made. The morphology of teats also appears to be heritable, with length, diameter and inter-teat distance all reported to be high to moderately heritable [68]. Most breeding/replacement programs should assess each piglet for teat number and location [62]. Production records in Sweden report 13% of gilts having at least one non-functional teat by five months of age which may indicate the need for teat damage prevention.

## 5. Piglet Fostering

Risk of mortality is high when piglet numbers exceed sow rearing ability and so fostering is widely adopted across pig industries. The process assists in re-homing excess piglets that are associated with increased sow prolificacy, in relation to available functional teats [69,70,71]. It can also be used to ensure litter uniformity, reducing weaning weight variability and subsequent impacts on slaughter management [72]. The process involves moving a piglet from one sow to another, and whilst aimed at solving excess piglet number and weight variation issues, piglet movement does not come without some cost. A change in environment, littermates and sow can prove to be of detriment for a fostered piglet, and can also upset the litter on to which it is relocated.

### 5.1. The Impact of Early Piglet Movement on Survival and Growth

Piglets establish a teat order in early lactation, and when this is not achieved in the first day, survival and growth are markedly reduced [73]. Heim et al. [70] demonstrated that on day one of lactation, litters of 100% biological piglets displayed a lower number of teat disputes than those composed of all fostered individuals (with fostering conducted at ~20 h). However, this did not translate to any differences in behaviour on subsequent days, and bore little impact on piglet growth suggesting that after fostering within 24 h of birth, competition at the udder is resolved quickly. This is further supported by Deen and Bilkei [33] who showed that weight gain was unaffected when piglets were fostered at 12 h of age. It would also appear that fostering has little impact on piglet survivability when the event occurs close to farrowing. Even when housed in farrowing pens which present a higher risk of mortality, fostered piglets have similar survival to biological piglets when the fostering event was conducted within 24 h of birth [74]. The authors of this study did conclude that this finding was likely explained by the fact that birth weight was higher in fostered piglets, which then begs the question; does piglet weight impact on fostering success?

### 5.2. Factors that Influence Growth and Survival of Fostered Pigs

Piglet size appears to be an important factor when trying to optimise growth and survival of a fostered individual. When fostered piglets are heavy and of high vigour they outperform biological piglets with regards to survival, but movement of average weight piglets results in lower survival rates [69]. This holds true even in pen systems where risk of mortality is greater [74]. Litters with variable birth weights tend to have a higher number of low (<900 g; small) rather than high birth weight piglets, leading to question the most appropriate strategy to foster low birth weight piglets [75]. This study, whilst finding a slight improvement in survival, demonstrated there was no impact on growth and in fact, teat disputes were higher when small piglets were grouped together. Huting et al. [76] reported a significant improvement in piglet growth performance from large piglets that were grouped in a mixed litter consisting of large and small piglets in comparison to large piglets fostered to a uniform litter, but the small piglets had greater growth when they were grouped with piglets of a similar size. Muns et al. [77] showed that litter survival was higher when low weights were grouped with high weights, but after adjusting for the proportion of smalls in the litter, there was no impact of foster piglet size on survival. However, others report the advantages of grouping small individuals together. When low birth weight piglets have high or average birth weight litter mates they generally miss more nursing episodes as they are outcompeted [33]. Similarly, Souza et al. [78] demonstrated that small piglets when grouped with large littermates missed more nursings in early lactation than when they were grouped with small littermates, but this did not translate to any differences in growth. This study did show that survival of small piglets was 100% when they were grouped with other small piglets, but was reduced to 83% when they were mixed with large littermates. Marcatti [79] found that small, fostered pigs into litters with other small fostered pigs had improved survival and weight gain relative to similarly sized biological piglets further highlighting the success of this strategy.

When small piglets are in large litters, competition for teats may be too high [80] and so litter size appears to influence the success of small piglet fostering. In a litter of 12 with large individuals, small piglets had a lower survival rate and reduced growth rate than those in a litter of eight with large littermates [33]. This was because when in large litter sizes, small piglets missed more nursing events than when in small litter sizes. Taken collectively, small piglets should be fostered to a sow with other small individuals, but when this is not possible, the litter size should be reduced by removal of the larger pigs.

There are issues that present themselves if we accept that small piglets do better when fostered into small litters. Firstly small piglets may not provide enough pressure to stimulate milk letdown events. Souza et al. [78] did indeed show that litters comprising completely of small fostered piglets tended to display reduced nutritive nursings on days three and five of lactation than those comprised of half large and half small piglets. This is most likely attributed to the fact that small piglets have reduced energy requirements. Given that the above-mentioned studies and others [46] show no difference or even improvements in weight gain when small piglets are grouped to one sow, there is little indication of reduced milk production of sows suckling litters consisting of small piglets. None the less, careful monitoring of small litters should take place. There is also some discussion at the production level as to whether small piglets require too much time investment from farrowing attendants with a low likelihood of survival. Thus, the teat space would be better allocated to a larger piglet with increased viability, and the small piglet euthanized. There is no available economic assessment of the two strategies to support this notion with regards to labour input at farrowing, but Widmar et al. [81] argues that feed and medication costs of these lightweight pigs is uneconomical during the grower and finisher stages. Any strategy that involves euthanasia of small piglets would give rise to ethical debate over whether it is appropriate to push for increased litter size without investing effort to keep what is born alive.

There is little evidence of any sex effects on fostering success mostly as none of the above-mentioned studies have examined the interaction, but a discussion is worthy. Litter sex ratio is statistically male biased with the sow investing more into sons than daughters [82]. However, female offspring have higher survival rates than males [83] and this may be further exacerbated in castrated males [84]. More specifically, male piglets have increased incidence of crushings, disease related mortality and impaired thermoregulation [82]. Females also grow faster than males, and this is especially true during transition times such as weaning [85]. Logically, if female piglets have an improved chance of survival and grow faster in times of stress, female fostered pigs may outperform male fostered pigs. This notion would benefit from research attention.

## 6. Disease Risk

Whilst creating litter uniformity through the “grading” of piglets helps to optimise survival and growth, this should be balanced with the risk of disease transfer. An outbreak of porcine reproductive and respiratory syndrome (PRRS) in an 1800 sow-farrow to feeder-pig herd was reported in a case study by McCaw [86]. Disease spread resulted in high incidences of late abortion, and increased born dead piglets and mummified foetuses. Piglets born alive had low viability and treatments with various antibiotics were unsuccessful in reducing mortalities. At the commencement of the outbreak, “grading” of piglets was common and so piglet movement from one litter to another was high. This fostering did nothing to control the disease, and the authors mention that it may have assisted in the spread of the virus. To minimise disease transfer, the fostering protocol adopted (known as McRebel protocol) involved little piglet movement: completed in the first 24 h, piglet movement was contained within one room, filling sows to udder capacity, whilst disregarding the size and sex of the piglets [86]. Implementation of McRebel fostering resulted in a reduction in preweaning and nursery mortality, and improvements in piglet weaning weight. Whilst PRRS may not be a direct disease threat to all countries or farms, this case study was able to demonstrate that minimal piglet movement reduces disease spread between farrowing crates, rooms and sheds. 

### 6.1. Piglet Movement after 24 h

The above would suggest that as long as fostering is conducted within the first 24 h, there is limited impact on litter integration. In a study that examined the effects of piglet movement at older ages it was shown that for those moved at either two, four or seven days of age suckling success was lower, vocalisations were more frequent and they were the target of more sow aggression than those moved at less than 24 h of age [87]. Similarly, when moved at six days of age, fostered piglets only achieved a weaning weight 75% of biological individuals so movement later in lactation also impairs growth [88]. Interestingly, this study did demonstrate that older fostering resulted in a better adaptation to weaning (with fewer fights and injuries), presumably through socialisation effects.

Traditionally, older piglets with impaired growth were shown to display improved growth when removed from their litter and fostered to a younger, similarly sized litter [80]. This will now be referred to as “cross-fostering”. More recently, some have questioned the effectiveness of cross-fostering and the resulting continual piglet movement. This is largely based on the idea that piglets develop teat fidelity (i.e., are loyal to a specific teat) in early lactation, with repeated piglet movements potentially proving to be disruptive to this notion. Several studies have found negative associations with excessive cross-fostering of piglets including disruption to nursing episodes, increased fighting between piglets at nursing resulting in more injuries, affecting growth rates of both resident and fostered piglets [89,90,91,92]. Alterations in the maternal behaviours of foster sows have also been noted, with increased aggression towards piglets and fewer milk let-down events when cross-fostering occurs throughout lactation [89,93]. The effect of continual cross-fostering on growth impairment is most prominent at weaning with reports of up to a 25% reduction in weaning weight when compared to those with no movement after 24 h [94,95].

### 6.2. Strategies to Relocate Piglets after 24 h

To balance the fact that the movement of piglets later in lactation is disruptive to biological litters and sows but to effectively manage piglets failing to thrive or during events such as sow death or agalactia, an alternate strategy is required. Utilising nurse sows has become a more common practice not only to deal with older piglet movement, but to provide udder space when the number of piglets born exceeds the available number of teats. A nurse sow is one that is cleared of her biological litter and is then replaced with an entirely new litter of piglets [95]. The process can either be direct (older piglets are placed on to the weaned nurse sow) or involve a staggered approach (“bump”; the nurse sows receives large, strong five to ten day old piglets, and their sow receives young piglets or newborn piglets without teat access) [96]. Even though nurse sow usage may appear an attractive option, care should be given to manage the increased piglet movement and so risk of disease. The extended lactation of nurse sows could potentially result in sow welfare breaches as it will result in an increased period of confinement within a crate and little respite from older piglets [97]. No differences in physiological indicators of stress have been detected in nurse sows delivering a 40 day lactation [98], and so perhaps this is a suitable maximal lactation length. It is logical to hypothesise that the extended lactation will result in changes in the subsequent reproductive output of nurse sows, and indeed, an increase, in litter size has been reported in these sows [99,100]. A less commonly studied or utilised approach is double nursing, in which a sow nurses her own litter for half the day, and another litter of excess newborn or ill-thrift piglets for the remainder [101]. Double nurse sows were actually shown to display a 2% reduction in piglet mortality when compared with single nurse sows. However, the authors note this treatment effect was most likely explained by the selection of good mothers and litters for the double nurse treatment. No piglet growth data were presented, but presumably if litters were only nursed for half a day these would have been reduced. Further work is required before recommendations on this strategy can be made. Artificial rearing is another means to deal with excess or weak piglets, and whilst similar growth rates are achieved [102], some have demonstrated that there are behavioural indications of poor welfare in such systems [103].

### 6.3. Conclusions and Recommendations

Taken collectively, the following points should be considered when making piglet fostering decisions. Colostrum is crucial for survival and there are immune factors best absorbed from the birth sow. Where there may be a risk that sow colostrum production will not meet piglets needs (extremely high numbers of piglets born, or gilts/low condition sows), piglets may need to be fostered to a farrowing sow in order for the piglets to receive adequate amounts, and this should occur prior to gut closure (24 h). However, this event should be rare and almost all piglets should be maintained on their birth sow and colostrum intake managed. Colostrum quantity and quality are best around farrowing, thus first born, big piglets often get high amounts whilst small, later born piglets may not get enough. Strategies should be employed to facilitate even colostrum intake of litters, or at least more than 200 g to ensure survival prior to fostering. Given this, in most cases fostering should occur at 12–24 h after farrowing. After this point, piglet movement should be avoided and alternate strategies such as the use of nurse sows employed.
